# The Role of Features Types and Personalized Assessment in Detecting Affective State Using Dry Electrode EEG

**DOI:** 10.3390/s20236810

**Published:** 2020-11-28

**Authors:** Paruthi Pradhapan, Emmanuel Rios Velazquez, Jolanda A. Witteveen, Yelena Tonoyan, Vojkan Mihajlović

**Affiliations:** imec The Netherlands/Holst Centre, 5656 AE Eindhoven, The Netherlands; paruthi.pradhapan@asml.com (P.P.); emmanuel.riosvelazquez@imec.nl (E.R.V.); jolanda.bax-witteveen@imec.nl (J.A.W.); ytonoyan@sissa.it (Y.T.)

**Keywords:** human affect, valence, arousal, EEG, machine learning, dry electrodes, wearable EEG

## Abstract

Assessing the human affective state using electroencephalography (EEG) have shown good potential but failed to demonstrate reliable performance in real-life applications. Especially if one applies a setup that might impact affective processing and relies on generalized models of affect. Additionally, using subjective assessment of ones affect as ground truth has often been disputed. To shed the light on the former challenge we explored the use of a convenient EEG system with 20 participants to capture their reaction to affective movie clips in a naturalistic setting. Employing state-of-the-art machine learning approach demonstrated that the highest performance is reached when combining linear features, namely symmetry features and single-channel features, with nonlinear ones derived by a multiscale entropy approach. Nevertheless, the best performance, reflected in the highest F1-score achieved in a binary classification task for valence was 0.71 and for arousal 0.62. The performance was 10–20% better compared to using ratings provided by 13 independent raters. We argue that affective self-assessment might be underrated and it is crucial to account for personal differences in both perception and physiological response to affective cues.

## 1. Introduction

The use of physiological signals is a preferred choice for objective assessment of emotions in humans as it is closely associated with processes emanating from both the central nervous system and the autonomic nervous system. Moreover, physiological effects are in most cases involuntary and cannot be manipulated or masked easily. Given the role that the limbic system plays in emotions, brain activity monitoring is of interest in the field of emotion recognition [1]. Reliable monitoring of limbic activity requires the use of technology that can monitor deep brain structures, e.g., magnetic resonance imaging (MRI). Although functional MRI studies are used to assess the emotional state of a person [2], they can bias subjects’ emotional state as he/she is placed in an environment that is quite different from a typical daily setting and that can per se impact the emotional state [3,4]. Therefore, using non-invasive surface electroencephalography (EEG) is currently seen as the most appropriate measurement modality for characterizing emotional processes [5,6,7]. It does not require complex measurement equipment or invasive procedures and allows data acquisition in natural settings. The main drawback of surface EEG is the low depth of penetration, making it difficult to directly capture the activity of the limbic system. However, modulation of the limbic activity affects also the activity of the cerebral cortex to a certain degree [8], making it possible to use EEG as a window to capture the emotional state of a person.

Although EEG allows users to act in a daily life manner, most research on emotions has been done with non-portable EEG systems while exposing users to a set of prescribed stimuli. This makes it difficult for the user to form an emotional bond due to either content type selected as stimuli or time limitations imposed [9]. The limitations in terms of the use of cumbersome EEG acquisition system has resulted in users being constrained toward full emersion into an emotional experience, while the reaction to stimuli, such as audio or pictures [10,11,12], was often missing or skewed. This led to low reproducibility and high variance in results under different acquisition protocols and stimuli used. Considering the absence of data cleaning algorithms and use of distinct feature types, it is not a surprise that coherent results in emotion characterization using surface EEG are missing.

One of the most frequently investigated features for emotion estimation is the approach/withdrawal model, which is based on the relative difference in alpha activity between the left and right hemispheres of the brain [13,14,15,16,17,18]. Per this model, relatively greater frontal activity (activity here is inferred as the inverse of alpha power) corresponds to a greater tendency to respond to affectively positive stimuli (i.e., approach) whereas, relatively greater right frontal activity indicates increased tendency of response to affective negative stimuli (i.e., withdrawal) [19,20]. Since then, asymmetry features have been used extensively for emotion recognition by various researchers with varying success [21,22,23]. However, contradictory results are also available showing that lower frequency bands (delta/theta) are modulated by emotional stimuli but alpha (and beta) are not. This suggests inclusion of different frequency band activity in emotion elicitation analysis. Recent studies have also shown that complexity and non-linear properties of the signal can be useful to characterize EEG changes due to emotional stimuli [24,25]. Fractal dimensions [26,27] and multiscale entropy (MSE) [28,29] have been shown capable of distinguishing emotional valence and affects, respectively.

The overall goal of our research is to accurately and reliably classify induced emotions from EEG, monitored in a convenient way, by using state-of-the-art features and advanced machine learning approaches. To minimize the impact of an EEG monitoring system on subjects, we introduced a wearable EEG headset system that does not require the use of conductive gel to capture EEG. It provides enough comfort to the user and enables free movement during the recording. This facilitates unimpaired engagement of subjects into emotional experience. Emotions are triggered by video watching, given that the combination of visual and audio cues was the most pronounced in eliciting emotions as it indicated the strongest activation in the emotion-arousal network, as shown by Baumgartner et al. [30]. Movie clips are used in this study as they evoke strong emotions [31,32,33,34] and are more natural than subject-elicited emotions (i.e., recollecting emotional experiences from memory). Furthermore, due to relatively long duration, they allow for emotion to be fully expressed [34].

As considerable variability is expected in the prediction accuracy depending on the features set used and method applied, we introduced a systematic approach. Besides spectral features per EEG location and differential (symmetric) features, we also included MSE to explore the impact of those on emotional state recognition. We evaluate the effect of feature importance and relevance of subjective scores on prediction performance. We propose not only an efficient emotion classification paradigm, but also explore the effect of various factors in the prediction. This includes evaluating differences between perceived emotions (i.e., emotions the movie clip was designed to evoke) and induced emotions (i.e., the actual emotions induced in subjects) [35]. Perceived emotions refer to intellectual processing, such as perception of an intended or expressed emotion, whereas induced emotion refers to the actual emotions felt by the subject while observing the intended/expressed emotional stimuli, which is related to emotional self-regulation [36]. The relationship between perceived and induced emotions is extensively described for music [37,38,39], but seldom studied for movie-based emotion perception. In this study, we evaluate the subjective assessment of the emotional state by subjects participating in the EEG study, to those from independent raters, who provided arousal and valence scores for the emotional content of each movie clip. This exploration was aimed at clarifying the effect of relying on personalized induced emotion rather than more objective perceived emotion as a ground truth.

By doing such a systematic analysis we contribute to understanding the limitations of predicting human emotions and indicate the sources of the constraints. Further, the limitations on the subjective assessment of emotions are explored. In addition, the outcome of this study should provide valuable information on the aspects that need to be addressed while designing both experimental paradigms and product solutions for accurate emotion recognition.

The paper is organized as follows. Section 2 provides information about the acquisition setup, protocol used and data analysis pipeline. Results are presented in Section 3, introducing information on subjective and independent raters, signal quality, and classification performance. Main contributions are discussed in Section 4, followed by a conclusion section.

## 2. Materials and Methods

### 2.1. Data Collection

#### 2.1.1. Subjects

This study was approved by an imec internal ethical committee. All the subjects were recruited on a voluntary basis. They were informed about the experimental evaluation beforehand and signed an informed consent. Data was collected on 20 subjects (age: 32.30 ± 7.17 years; 15 males) using imec’s 8-channel EEG headset and acquisition software while performing the movie watching paradigm. The participants were also given the choice to stop the experiment at any point during the measurements. All subjects participating in the study declared that they do not have known neurological diseases or other medical conditions, conforming to the inclusion criteria.

Since the movie clips used in this study did not have prelabeled data for arousal and valence metrics, an independent movie rating session was performed on an additional 13 participants to obtain scores for arousal and valence of each movie scene used in the affective movie dataset. The mean scores obtained from the independent raters were used as the perceived emotions score. Subjects who participated in the movie rating session were not allowed to participate in the data collection experiments.

#### 2.1.2. Acquisition Setup

**Figure 1:**
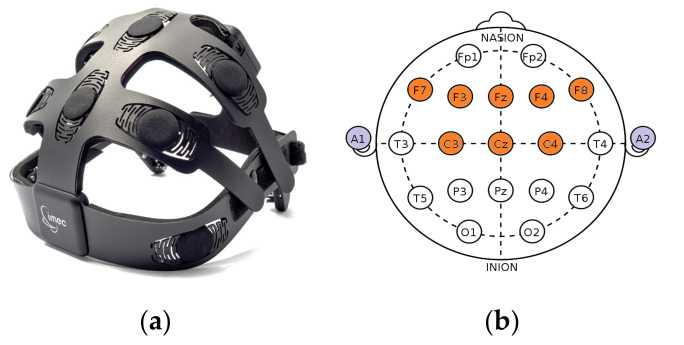
(**a**) IMEC 8-channel electroencephalography (EEG) headset and (**b**) electrode positions on the scalp based on the imec wireless headset.

For emotion and cognition recognition, an 8-channel headset with electrode configuration covering the frontal and central regions of the scalp was designed by imec. The 8 channels are located at F3, F4, Fz, F7, F8, C3, C4, and Cz of the International 10–20 electrode positioning system. Patient bias is located at left mastoid (A1) and reference channel at right mastoid (A2). Dry conductive polymer electrodes with silver/silver-chloride coating are integrated into the headset, making it easy to set up and convenient for the users during the measurements. Since the EEG headset is a highly integrated system with data transmission over Bluetooth [40], the participants were not infringed of natural movements during the measurements. The system is designed to continuously measure contact impedance at each channel, thereby allowing the possibility for data quality assessment during analyses. The design and headset configuration are depicted in Figure 1.

A software developed at imec was used to acquire the EEG data, visualize it in real-time, and render movie clips and the PANAS questionnaire. The software facilitated inspection of EEG signal quality required for optimal mounting of the headset and enabled synchronization of displayed content with the acquired EEG. The data is stored in a Hierarchical Data Format 5 (HDF5) for later analysis.

#### 2.1.3. Protocol Description

**Figure 2:**
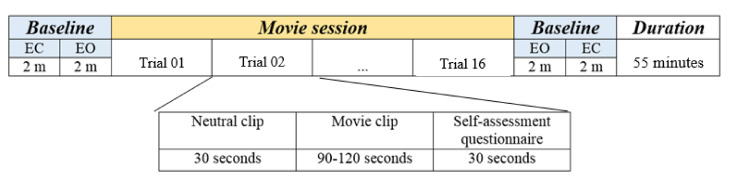
Timeline for baseline and affective measurements during one session. EO—eyes open; EC—eyes closed. Each trial consists of a neutral clip, movie clip, and s-PANAS questionnaire.

All the experiments were performed in a laboratory setting. Participants were seated comfortably, about 1.5 m away from a 40-inch LCD/LED television, which was used to play the arousal/valence elucidating content. Each session began with a baseline measurement, where the subjects performed eyes open and eyes closed tasks for 2 min each and were repeated at the end of each measurement session. Movie clips from popular movies, known to elucidate excitement/calmness (i.e., arousal) and positive/negative emotions (i.e., valence) from previous literature [34], were played in sequential order. In total, 64 movie clips of about 90–120 s were played during the measurements (see Appendix A: Movie Clips for the complete list). Prior to each movie clip, a neutral video of 30 s duration was played to allow the subject to return to the baseline emotional state, as suggested in the literature [34]. Due to the long duration and to avoid fatigue, the measurements were divided into four sessions each consisting of 16 clips each. The participants had two short breaks of 10–15 min after first and before the last session and a 30-min break after the second session, during which the EEG headset remained mounted on the subject’s head. Participants were strongly encouraged not to adjust the positioning of the headset. The experimental sequence is depicted in Figure 2.

After each movie clip, a subjective assessment questionnaire was presented to the subjects to rate his/her feeling of arousal and valence. To achieve a balance between resolution of emotional state and quick response, the s-PANAS [41] was chosen as means of subjective assessment in our experiments. The subjective ratings of the participants formed the scores for induced emotions.

### 2.2. Data Analysis

**Figure 3:**
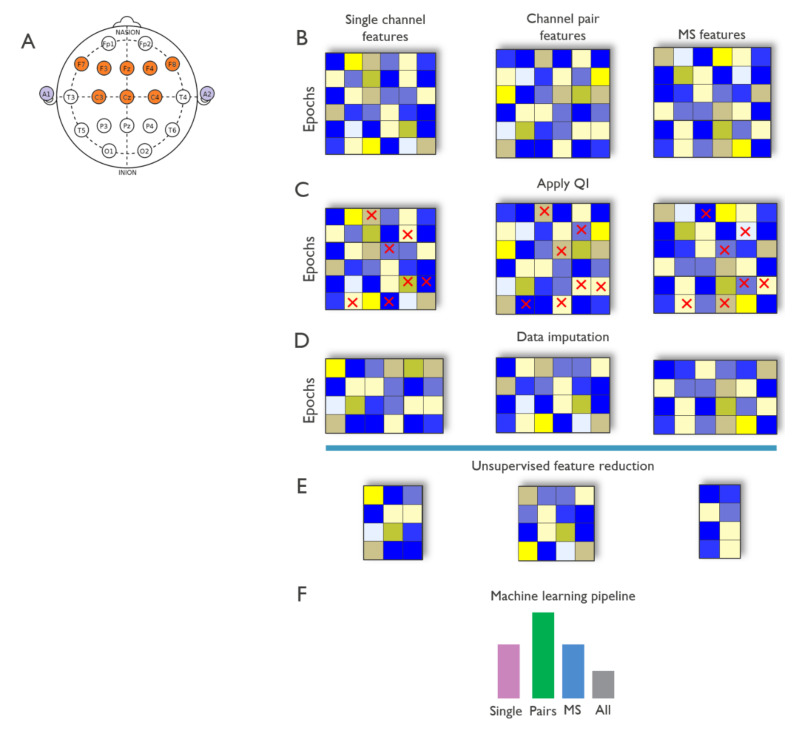
Block diagram of data analysis. (**A**) Electrode positions on the scalp based on the imec wireless headset. (**B**) Per participant, EEG feature sets were extracted from single channels (i.e., F8), channel pairs (i.e., F3 and F4) and multiscale (x, y, and z channels), for 8-s epochs across the entire movie watching recording. (**C**) A data quality indicator was applied to exclude poor quality epochs. (**D**) To reduce data loss due to signal quality, the feature sets were subjected to missing-value imputation. The imputed high-quality feature sets of each participant were integrated into a pooled dataset (B–D were performed per subject independently). (**E**) Unsupervised feature reduction based on variance (PCA) and correlation among features was applied to each integrated feature set to reduce feature redundancy. (**F**) The uncorrelated variance retaining feature sets were then used to train and validate random forest classifiers for arousal and valence using k-fold cross-validation and nested supervised feature selection. Models combining all three feature sets were also evaluated for comparison (E–F were performed in the integrated dataset).

The data analysis pipeline follows the recommended state-of-the art affective system architecture [42,43] and consists of the following steps: data preprocessing, signal quality estimation, extraction of features relevant for emotion detection, feature importance estimation, and valence and arousal classification. The first three steps were implemented in Matlab (The Math Works, Inc., Natick, MA, USA, *MATLAB*. Version 2018a), while the last two are coded in Python. Three feature sets were evaluated for their ability to predict arousal and valence: single channel (e.g., F8), differential (e.g., C3 and C4), and multiscale entropy features (derived over F3, F4, Fz, F7, and F8). The complete pipeline is shown in Figure 3.

#### 2.2.1. Data Preprocessing

A band-pass filter (5th order Butterworth) in the 2–45 Hz frequency band and a 49–51 Hz notch filter (5th order Butterworth) to remove the powerline interference were applied. After filtering, EEG data during each movie sequence recorded was segmented based on the timing information captured along with EEG data. The length of each segment depended on the length of the corresponding movie clip. Further, EEG data during each movie segment was divided into 8-s epochs without overlaps to obtain independent feature set. Epoch duration was selected based on the work of Candra et al. [44], indicating that a window size between 3 and 10 s in length will produce consistent results in terms of classifying emotions. The feature set was computed on the 8-s epochs for all movie segments, given that these epoch durations resulted in a stable feature set for both signal quality estimation and affect detection in the initial empirical evaluation.

#### 2.2.2. Signal Quality Estimation

The signal quality indicator is based on a set of time and frequency domain features optimized to detect noise from a variety of sources like environmental noise, eye artifacts, and motion artifacts. The signal quality indicator uses a pragmatic approach, which is based on thresholds to determine if an epoch is of good or bad signal quality. It uses statistical features and is captured within an EEG epoch. More details on the estimator and its performance are reported here [45]. The signal quality indicator gives an output from 0 to 1, 0 corresponding to the highest quality and 1 to the lowest quality. Signal quality is computed for each headset channel at each 8-s epoch. Only epochs with a quality index equal to 1 (certain of low quality) were removed from further analysis.

#### 2.2.3. Feature Extraction

A set of temporal and spectral features relevant to extracting valence and arousal metrics were computed. Temporal features include approximate entropy, sample entropy, and fractal dimension. Spatial features are power spectral density in each frequency band (delta, theta, alpha, beta, and gamma) and differential entropy. The frequency band used included: delta (1–4 Hz), theta (4–8 Hz), alpha (8–13 Hz), beta (13–30 Hz), and gamma (30–45 Hz). Those features were extracted for each epoch. Typically, three different input parameters were required for computing approximate and sample entropy: length of the sequence to be compared (m), tolerance threshold for accepting similarity criterion (r), and length of total data sequence (N). Based on the literature [46,47], we set the input parameters to m = 2, r = 0.2 × standard deviation, and N = data duration (in seconds) × sampling rate. The input parameters for computing Higuchi’s fractal dimension were determined based on the recommendations by Doyle et al. [48].

Aside from these more common features, features derived from the framework of multiscale entropy were estimated. Since biological systems operate on multiple time and space scales and therefore their complexity was also multiscaled, multiscale entropy has the potential to better describe brain activity changes related to emotional stimuli. Multiscale entropies as complexity related metrics could discriminate signals generated either by different systems or by the same system under different conditions. Entropy features obtained from multiscale decomposition of EEG recordings have shown to discriminate between different cognitive [49] emotional states [50,51,52] and hence are explored in this study.

Rényi entropy (RE) [53] was used in this study as several successful EEG-based clinical applications are reported in the literature [54,55] and usefulness in emotion estimation [56,57]. A nonparametric estimation of RE was chosen.

We estimated RE in a non-parametric way by computing it from the data kernels [58]. The results show that signals were considered complex if there was a long-range correlation across multiple time scales, i.e., they should be neither random nor regular [59]. The previously cited results computed entropies as a measure of regularity (irregularity). In order to use entropies as a complexity measure, the above-mentioned multiscale correlation should be taken into account by computing entropies on multiple time scales. For the data scaling, empirical mode decomposition (EMD) [60] and its multivariate version (MEMD) [61] were used. EMD is a time frequency technique to decompose a given signal into amplitude (or frequency) modulated counterparts, which are called intrinsic mode functions (IMFs). Using EMD, the observed signal with N samples was decomposed into a_l_ = log_2_N IMFs: *Signal* = *IMF*_1_ + *IMF*_2_ + … + *IMF_l_*. The first IMF represents the highest frequency component and the following ones captured lower and narrower bands. The last component, *IMF*_l_, is the trend in the signal and is usually omitted from further analysis. The MEMD aligns similar frequency bands of multiple channels, thus, providing an assessment of their possible interdependence (mode alignment property). Therefore, the algorithm to compute MSE is as follows:

- Decompose the given signal into intrinsic mode functions (IMFs) using MEMD;
- Compute entropy for each IMF.

To compute the asymmetry indices, the natural log transformation of power within specific frequency bands was computed. The natural log transformation technique was used to normalize the distributions of power values. Differential asymmetry was then computed as the difference of power in individual frequency bands between the right and left hemispheric channels. Rational asymmetry is another feature used in asymmetry studies and is a variation of differential asymmetry. It uses the ratio, instead of difference, when the natural logarithm of spectral power is computed between the symmetrical electrode pairs.

#### 2.2.4. Performance Evaluation

Three EEG feature sets were evaluated to assess their ability to estimate arousal and valence: single channel, differential and multiscale entropy features. We evaluated the performance of these features independently and in a combined model.

Application of the EEG signal quality indicator resulted in a significant number of epochs excluded from further analysis. To reduce data loss due to signal quality, the feature sets were then subjected to a missing-value imputation as follows: for a given movie, if an epoch had more than 50% of the channels with bad quality, the entire epoch was removed. Subsequently, for each column within the movie, if a column (e.g., ‘Sample_entropy_Cz’) had ≤ 20% missing values throughout the movie, the missing values were imputed using the column mean. The subjects’ imputed high-quality feature set was then combined into an integrated dataset per feature type.

To identify variant and uncorrelated features, we performed a principal component analysis (PCA) based feature reduction analysis to identify the highest correlated features (Pearson r > 0.7) to the principal components that describe at least 95% of the variance in the pooled dataset. Then, a second feature reduction step based on correlation was applied to identify feature pairs to be removed to reduce pairwise correlations. A correlation coefficient of 0.95 was used as a cut-off.

The variance-retaining and non-correlated features were used to build random forest classifiers to predict arousal and valence, using either single channel, differential, or multiscale features as follows: we used a 5-fold cross-validation to build and evaluate RF models. In the training set, features were ranked based on mutual information between the features and the arousal and valence (scores). The 10 top-ranked features were used to fit an RF model. The trained models were then evaluated in the test set. Given that features of the same subject can be a part of both training and validation set, the presented results were subject dependent.

A fourth set of models combining the features selected based on mutual information for single channel, differential and MSE features were built for comparison.

F1-score, a weighted average of precision and recall commonly used for unbalanced datasets, was used as performance measure. For comparison, independent rater scores were used as well to train random forest classifiers for valence, for the different feature groups.

A rank sums Wilcoxon test was used to evaluate whether the performance of a feature group was significantly different. One-sided *p*-values smaller than 0.05 were considered statistically significant.

All analyses were performed using the Python (3.7.3) scikit-learn package (0.20.3) and scipy (0.2.1).

#### 2.2.5. Subjective Valence and Arousal Scores

The subjective scores for valence and arousal were computed from the s-PANAS questionnaire responses obtained from the participants at the end of each movie clip. The valence scores were computed by subtracting the cumulative scores of negative emotions (i.e., upset, hostile, ashamed, nervous, and afraid from the s-PANAS) from the cumulative score of positive emotions (i.e., alert, inspired, determined, attentive, and active from the s-PANAS), as shown in the equation below. A positive score indicated “positive” valence and vice versa for “negative” valence.

$$S_{\mathrm{Valence}}=(S_{\mathrm{Alert}}+S_{\mathrm{Inspired}}+S_{\mathrm{Determined}}+S_{\mathrm{Attentive}}+S_{\mathrm{Active}})-(S_{\mathrm{Upset}}+S_{\mathrm{Hostile}}+S_{\mathrm{Nervous}}+S_{\mathrm{Ashamed}}+S_{\mathrm{Afraid}})$$

Similarly, the arousal scores were computed by adding the scores for each emotion on the s-PANAS list. The minimum score achievable was 10 and maximum 50. A midline threshold, set at 30, distinguished between ‘low’ and ‘high’ arousal.

$$S_{\mathrm{Valence}}=S_{\mathrm{Alert}}+S_{\mathrm{Inspired}}+S_{\mathrm{Determined}}+S_{\mathrm{Attentive}}+S_{\mathrm{Active}}+S_{\mathrm{Upset}}+S_{\mathrm{Hostile}}+S_{\mathrm{Nervous}}+S_{\mathrm{Ashamed}}+S_{\mathrm{Afraid}}$$

## 3. Results

### 3.1. Comparison between Subjects’ and Independent Rater’s Valence and Arousal Scores

**Figure 4:**
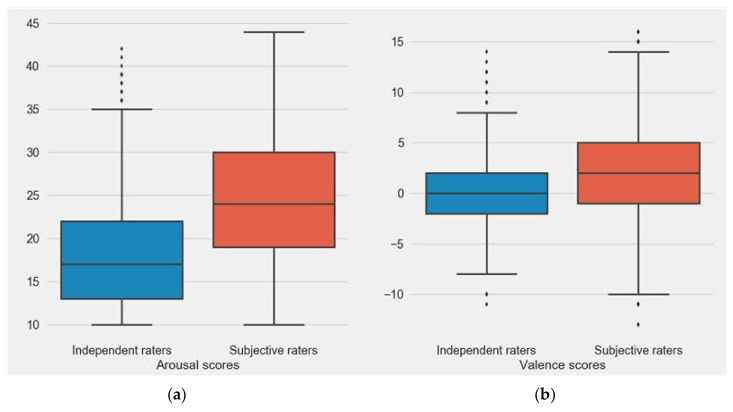
Distribution of arousal (**a**) and valence (**b**) scores between subjective and independent raters.

To determine the relation between perceived and induced emotion, a correlation analysis between subjects’ and independent raters’ was performed. A significant positive correlation was observed for both, arousal (r = 0.9346, *p* < 0.001) and valence (r = 0.9002, *p* < 0.001). Figure 4 shows the distribution of scores in the subjective and independent raters’ groups, respectively. The non-parametric Kolmogorov–Smirnov test revealed significant difference between the distribution of arousal scores from subjective and independent raters’ assessment (*p* < 0.001). Similarly, significant differences exist between the distribution of subjective and independent raters’ valence scores (*p* < 0.001). Significantly higher arousal and valence scores were observed in subjects who participated in the study.

### 3.2. Signal Quality Estimation

**Figure 5:**
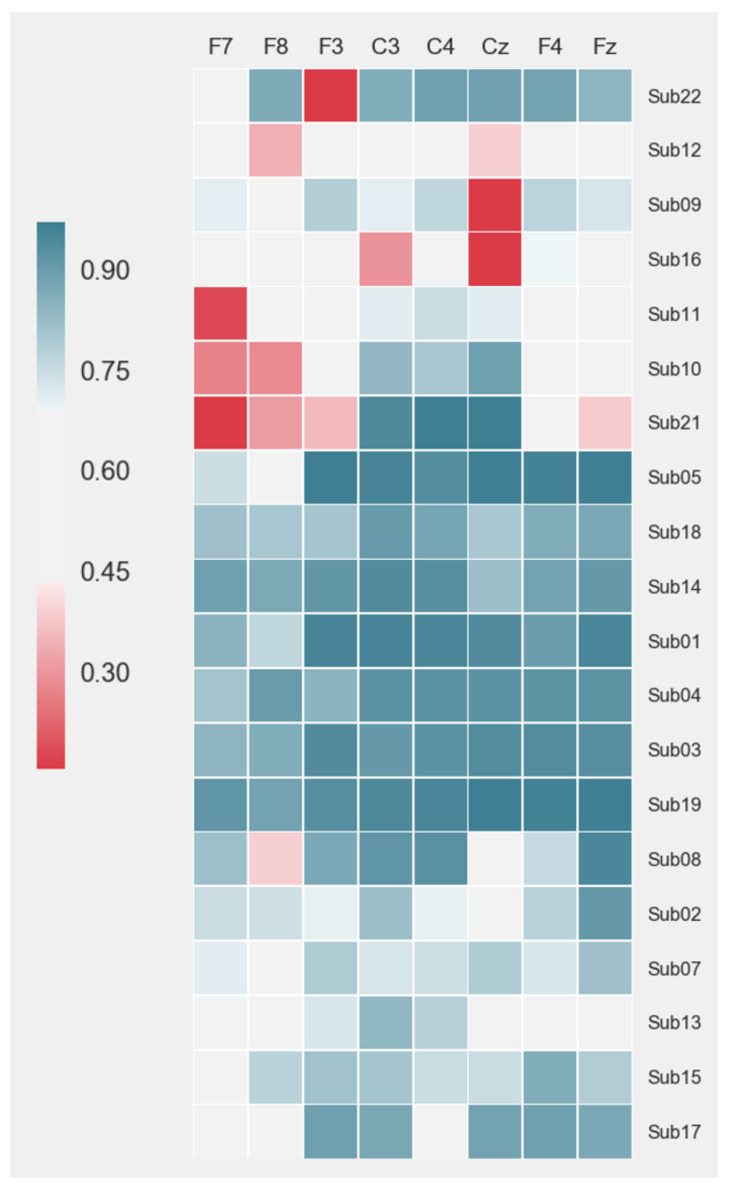
Percentage of good quality epochs per subject for each headset channel. Subjects were clustered from a low to high percentage of artifact-free epochs.

Applying the data quality indicator algorithm resulted in marking to 6736 out of 16,334 epochs as artifact-free data. The percentage of high-quality epochs for each subject is shown in Figure 5. Channels F7 and F8 were most affected by artifacts with the lowest average percentage of artifact-free epochs (F7: 69% ± 20% and F8: 67% ± 19%). The main cause was a strong presence of eye blink and eye movement artifacts in those channels. Channels C3, C4, and Cz, on the contrary, showed the highest percentage of artifact-free epochs across all subjects: (C3: 84% ± 14%; C4: 84% ± 12%; and Cz: 75% ± 28%). This is expected due to lower dominance of eye activity induced artifacts in those channels. Participants, such as subjects 12 and 16 showed lower data quality across all channels. Subject 19 showed the highest percentage of good quality data. The difference in data quality across subjects was mainly due to the limitations in the fit of the EEG headset. Although designed for medium-size heads (circumference 52–55 cm), subjects having larger or smaller head circumferences were not excluded from the study. As a result, not all dry electrodes in the headset had good enough contact with the scalp.

### 3.3. Features and Classification Performance

**Figure 6:**
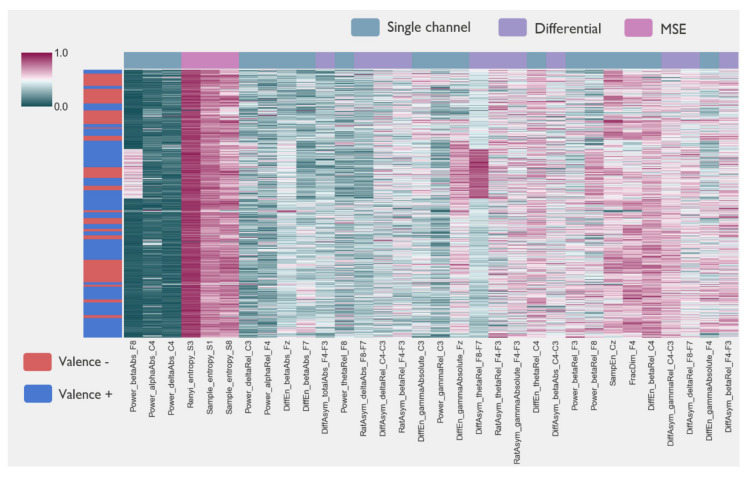
Single channel, channel-pairs, and multiscale entropy EEG features for a representative subject after the feature reduction step. Rows correspond to EEG epochs for the entire recording (64 movies). The row colors (left) correspond to the valence scores assigned to the given epoch. Normalized feature values are shown in the cluster map.

**Figure A1:**
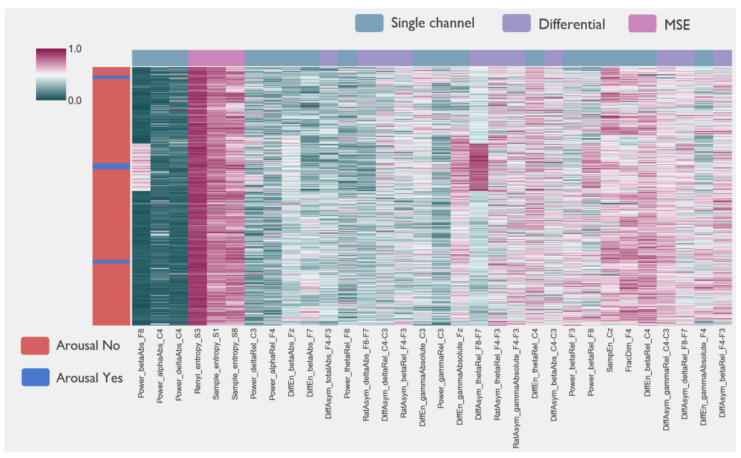
Single channel, channel-pairs, and multiscale entropy EEG features for a representative subject after the feature reduction step. Rows correspond to EEG epochs for the entire recording (64 movies). The row colors (left) correspond to the arousal scores assigned to the given epoch. Normalized feature values are shown in the clustermap.

Figure 6 shows the distribution of the different EEG feature sets over the entire recording for a representative subject. Only features left after feature reduction are shown. Features extracted from the same channels show a higher degree of redundancy and were clustered together, and similar feature types on adjacent channels. Multiscale entropy features showed a high degree of correlation and can be described with a limited number of scales. Overall, single features did not seem to be able to separate the epochs between positive and negative valence (see Figure A1 for an example on arousal).

To investigate the complementary value of single channel, channel pairs, and MSE features, and the relative importance of features in predicting emotional arousal and valence, we built RF models using either single channel, channel pairs, or MSE features and a combined model (single, pair and MSE features) for comparison.

**Figure 7:**
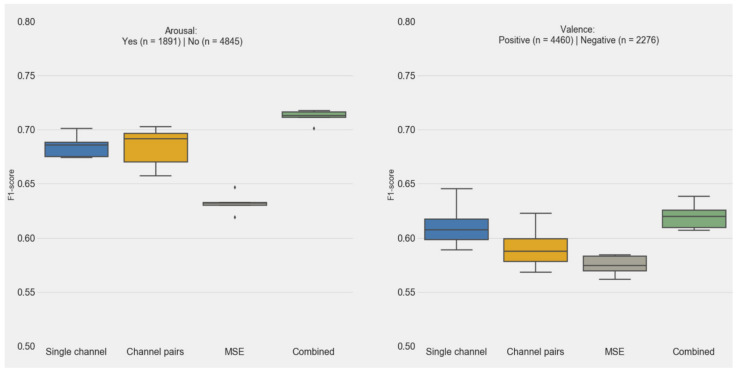
Five-fold cross-validation F1-scores for the prediction of emotional valence and arousal using either single-channel, channel-pairs, MSE features, or their combination. The asterisk sign represent score distribution outliers.

Figure 7 shows the distribution performances of the k-fold cross-validated random forest models for arousal and valence, for each feature set. For arousal, the combined model showed the highest F1-scores (mean = 0.70, CI, 0.69–0.72), compared to each individual feature set (0.016, 0.028 and 0.009, one-sided Wilcoxon-test *p* values for the comparisons with single-channel, channel-pairs, and MSE models respectively). Models trained with multiscales showed lower performances than models using single-channel and channel-pairs features (one-sided Wilcoxon-test *p* < 0.01).

For valence, the RF classifiers showed slightly lower performances than for arousal, but with the same trend: the combined models showed the highest F1-scores, along with the single-channel features (*p* = 0.17, for comparison combined models vs. single-channel models), while models using channel-pairs and multiscale features showed lower performances. The combined model showed significantly higher F1-scores compared to the channel-pairs and MSE (0.016 and 0.009, one-sided Wilcoxon test).

### 3.4. Subjective and Independent Rater Scores

**Figure 8:**
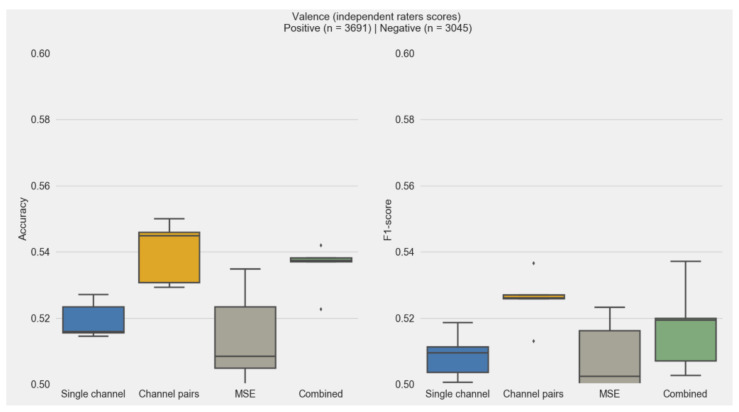
Accuracy and F1-scores for the prediction of emotional valence using the independent raters’ scores as labels to train the random forest classifiers. The asterisk sign represent score distribution outliers.

**Figure A2:**
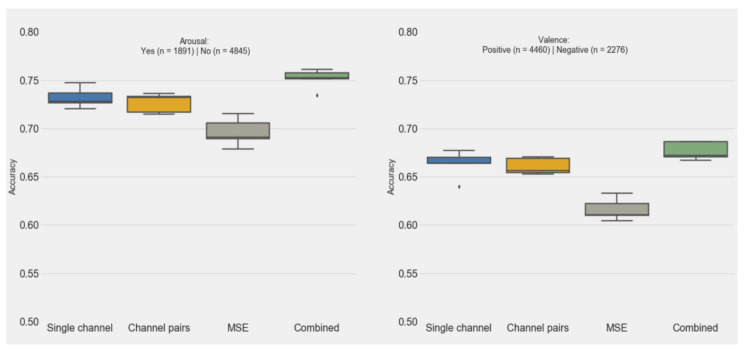
Five-fold cross validation accuracy scores for the prediction of emotional valence and arousal using either single-channel, channel-pairs, MSE features, or their combination. The asterisk sign represent score distribution outliers.

The emotional valence classification performance across different feature sets, when using independent rater scores instead of subjective scores, is shown in Figure 8. A feature performance comparison for arousal was not possible using the independent raters’ scores, as in all cases, the scores for arousal were below the threshold of 30 (s-PANAS scale midpoint), which resulted in a single label for all movies (i.e., not aroused). This is also illustrated in Figure A2. Compared to the scores achieved when using subjective scores, a substantial drop in F1-score can be observed (5–10%). Furthermore, the best performance was achieved when using channel pairs, in contrast to using all features.

## 4. Discussion

The main goal of this work was to evaluate the feasibility of using a dry electrode, wearable wireless EEG system to predict emotional states accurately utilizing advanced machine learning algorithms in a real-life scenario. Various features described in the literature, which include standard EEG time-frequency, asymmetry, and MSE features, as efficient means of classifying emotional states were evaluated in this study. The performance achieved, assessed using F1-score ranges from 0.62 (valence) to 0.71 (arousal). This is not far below the state-of-the-art approaches that use a more comprehensive setup with a much larger number of electrodes and lower impact of artifacts, due to the lower presence of movements and the use of conductive gel electrodes [62,63]. The analysis shows that using scores of subjects themselves instead of the mean scores of independent raters gives much better performance suggesting the importance of considering personalization both in the human affect assessment and classification performance. Finally, we emphasized the role of preparation and curing the data as an initial processing step to ensure only artifact-free features were used in the evaluation. Signal quality computation is a significant step to ensure only artifact-free data was used in the analysis. On average, 23% of the data acquired during the study were corrupt by artifacts and were imputed by means described in the Methods section. The magnitude of corrupt data can be attributed to the fact that the subjects could perform their natural response to visual cues, without restrictions on the kind of physical activity or emotions they could display, hence it resembles closer real-life applications. This ensures that the natural physical and emotional responses are not impeded due to the experimental setup, however, it resulted in larger data corruption than in the typical EEG user studies that involve movement and gesture restrictions (cf. [62,63]).

The classification performance when using MSE was lower than when using channel pairs, and individual channel features. Though unexpected and contrary to some of the recent results reported in the literature [64,65], this could be attributed to the interpersonal differences captured, stemming from both different natures of physiological responses and in having responses captured differently by the MSE components, thus preventing effective generalization. Nevertheless, combining all three different feature sets resulted in a performance boost, compared to using an individual feature set when applying machine learning. This confirms that emotional affect is a complex phenomenon that impacts EEG activity at a specific location, and the interaction of brain regions, in both linear and nonlinear aspects.

The overall performance of generalized models can be assessed as modest, given that the F1-scores of 0.63–0.72 for valence and 0.56–0.65 for arousal were achieved [62,63]. This confirms the state-of-the-art indicating that personalized models are required to further improve classification performance. Additionally, valence and arousal might have a fluctuating course while a person is watching a single movie clip, making it difficult to identify specific affect and a fixed brain response (i.e., feature values) corresponding to a single movie clip.

To differentiate between perceived and induced emotions we compared the classification performance based subjective scores of emotional ratings by the subjects themselves and ratings by subjects independent of the study protocol. It was observed that the subjective ratings of arousal and valence were significantly different than those of independent raters. This confirms the distinction between the induced and perceived emotional response while watching video material, previously established while studying affective states produced by music pieces [29,30,31,32,33,34,35,36,37]. Furthermore, this suggests the bias of participants assessing the affective state while wearing an EEG headset from the ones merely watching the movie clips. Having lower performance when using independent raters scores, indicates that subjective assessment of one’s affect might be less far from the true state, contrary to often raised concerns related to this matter. However, other aspects might have impacted the results, such as different conditions of the movie watching experience between the two groups, e.g., watching in a group vs. alone and no use vs. use of the EEG headset.

The study was done on a single type of affective stimuli and a limited number of both subjective and independent raters. Using other emotion eliciting modalities, e.g., pictures and audio, can provide complementary input and either confirm or dispute some of the hypotheses discussed. Introducing other sensing modalities can contribute to better classification performance and better understanding of human affect [66].

Although subjects were instructed to behave as “normal” as they could while watching movie clips, the procedure of mounting the headset and the wearing experience itself could result in a substantial bias towards participants. Together with the limited number of EEG channels, these are the most important limitations of the study that need to be addressed to provide a more complete view on how the human affect is represented in brain activity modulations. For completeness, we list the most important features in Appendix B.

## 5. Conclusions

By using convenient dry-electrode EEG we were able to capture the user reaction to affective movie clips in a naturalistic setting. (A) Symmetry, single-channel, and nonlinear features seem to have different roles in affect processing. A personalized approach as compared to a more generalized models and independent affect ratings resulted in a better performance. Together with accounting for noise and artifacts in the signal, exploring those aspects in an affect identification task while introducing different affective sources will lead to new insights in understanding the impact of affect on human brain activity.

## Figures and Tables

**Figure A3:**
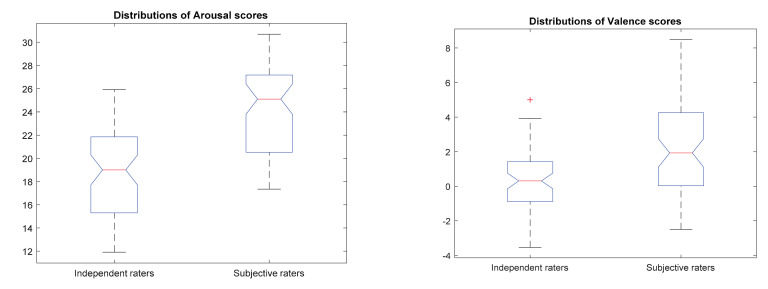
Distributions of the distribution of arousal and valence average scores per movie between subjective and independent raters. The plus sign denotes outliers in the score distributions.

## Appendix A. Movie Clips

Movie: 28 Days Later.

**Scene**	**Start Time**	**Stop Time**	**Description of the Start of the Scene**
**1**	00:09:05	00:10:09	Jim (Cillian Murphy) walks in the deserted streets of London with plastic bag in his hand
**2**	00:13:39	00:15:15	Starts with a close shot on Jim’s face in the church
**3**	00:42:33	00:44:55	The taxi starts ascending the trash dump in the tunnel
**4**	00:49:04	00:50:02	Inside the grocery store in the deserted gas station
**5**	01:08:29	01:09:24	Major Henry West (Christopher Eccleston) and Jim are walking in a corridor
**6**	01:34:58	01:35:46	Hannah (Megan Burns) is sitting in a red dress frightened in a room
**7**	01:36:09	01:37:58	A fight between the black soldier and a zombie
**8**	01:39:14	01:39:48	Jim opens the taxi’s door facing Major Henry West

Hotel Rwanda

**Scene**	**Start Time**	**Stop Time**	**Description of the Start of the Scene**
**1**	00:07:09	00:07:50	General Bizimungu (Fana Mokoena) and Colonel Oliver(Nick Nolte) are talking in the hotel’s garden (while tourists are in the pool in the background)
**2**	00:09:37	00:10:58	In the Paul’s (Don Cheadle) house, their son run into the living room frightened
**3**	00:23:04	00:24:21	Discussion between Paul and the Rwandan officer and he asks for Paul’s ID
**4**	00:51:04	00:54:33	French soldiers are checking tourists’ passportst
**5**	01:07:45	01:09:16	The hotel’s van is passing by is passing by burning houses at night
**6**	01:11:41	01:12:25	The hotel’s van is on the road in a foggy dawn
**7**	01:27:03	01:28:40	Rebels are dancing around the road waiting for the UN trucks
**8**	01:28:15	01:29:39	Rebels are hitting the refugees in the truck

Kill Bill Vol. 1

**Scene**	**Start Time**	**Stop Time**	**Description of the Start of the Scene**
**1**	00:05:46	00:06:46	Uma Thurman is fighting with Vernita Green (Vivica A. Fox)
**2**	00:58:50	01:01:00	The Japanese gangs are sitting around a black table
**3**	01:10:13	01:11:40	Japanese gangs are drinking in a room
**4**	01:15:02	01:17:07	Gogo Yubari (Chiaki Kuriyama) starts fighting with Uma Thurman
**5**	01:18:24	01:22:50	The fight scene of Uma Thurman and Japanese fighters in black suits
**6**	01:24:43	01:25:40	The fight scene of Uma Thurman and the Japanese bald fighter (Kenji Ohba)
**7**	01:28:48	01:30:53	The final fight scene between Uma Thurman and O-Ren Ishii (Lucy Lin)
**8**	01:02:10	01:03:35	Motorbikes are escorting a Mercedes in Tokyo streets

Love Actually

**Scene**	**Start Time**	**Stop Time**	**Description of the Start of the Scene**
**1**	00:12:19	00:13:57	Colin Frissell (Kriss Marchall) serves at the party
**2**	00:47:37	00:49:09	Aurelia (Lúcia Moniz) is bringing a cup of coffee for Jamie Bennett (Colin Firth) in the garden
**3**	01:18:28	01:20:33	The jewelry salesman (Rowan Atkinson) is wrapping a necklace
**4**	01:23:45	01:27:04	Colin Frissell arrives at Milwaukee
**5**	01:43:19	01:44:57	The old lady opens the door and surprises by seeing the prime minister (Hugh Grant)
**6**	01:51:38	01:54:14	School’s Christmas concert starts
**7**	01:58:15	02:05:00	Jamie Bennett arrived at Portugal
**8**	00:28:26	00:29:52	Daniel (Liam Neelson) and Sam (Thomas Sangster) are sitting on a bench

Mr. Bean’s Holiday

**Scene**	**Start Time**	**Stop Time**	**Description of the Start of the Scene**
**1**	00:05:39	00:07:22	Mr. Bean (Rowan Atkinson) takes a taxi at the train station
**2**	00:10:41	00:12:59	Mr. Bean is being served in a French restaurant
**3**	00:31:52	00:33:50	Mr. Bean is trying to raise money by dancing and imitating singer’s acts
**4**	00:37:33	00:39:19	Mr. Bean rides a bike on a road
**5**	00:40:37	00:42:35	Mr. Bean tries to hitchhike
**6**	00:45:45	00:47:15	Mr. Bean wakes up in a middle of the shooting of a commercial
**7**	01:08:04	01:09:02	Dressed as a woman tries to get into the theater with a fake ID
**8**	01:11:35	01:13:22	Mr. Bean is changing the projecting movie to his webcam videos

Ringu

**Scene**	**Start Time**	**Stop Time**	**Description of the Start of the Scene**
**1**	00:05:32	00:08:15	School girls are frightened by hearing the ring tone
**2**	00:27:39	00:29:38	Reiko (Nanako Matsushima) watches the video alone in an empty room
**3**	00:59:39	01:01:52	Reiko sees the past in black and white
**4**	01:19:10	01:21:48	Reiko is descending into the well
**5**	01:25:24	01:27:54	Ryuji (Hiroyuki Sanada) is writing at home and he notices that the TV is on and showing the terrifying video
**6**	01:29:56	01:31:55	Reiko seats on the sofa in her house
**7**	01:12:05	01:14:48	Reiko and Ryuji are pushing the well’s lead
**8**	00:48:20	01:49:42	Reiko is sleeping in her father’s house

Saving Private Ryan

**Scene**	**Start Time**	**Stop Time**	**Description of the Start of the Scene**
**1**	00:04:29	00:06:08	Start scene of the approaching of boats with these words appear “6 June 1944”
**2**	00:06:09	00:08:15	Landing on the Omaha beach
**3**	00:09:33	00:12:56	Combat scene on the beach
**4**	02:13:38	02:14:23	The sniper is praying when he is on a tower
**5**	00:18:51	00:21:38	The commander is looking into a mirror to see the source of the gunfire in a combat scene
**6**	02:26:05	02:27:21	The combat scene where Capt. John H. Miller (Tom Hanks) was shot
**7**	00:56:04	00:57:25	While they are looking for private Ryan, by accident a wall collapses and they face a group of German soldiers on the other side of the destroyed wall
**8**	01:20:00	01:20:45	Group of soldiers are walking on a green field

The Pianist

**Scene**	**Start Time**	**Stop Time**	**Description of the Start of the Scene**
**1**	00:00:24	00:02:09	Warsaw in 1939 (black and white shots)
**2**	00:21:10	00:22:34	Szpilman (Adrien Brody) is playing in a restaurant
**3**	00:24:28	00:25:54	Szpilman walks in the streets of Warsaw
**4**	00:32:57	00:34:11	A crazy man and children on the street
**5**	01:56:12	01:58:13	Szpilman (with long hair and beards) tries to open a can
**6**	00:44:34	00:47:07	Jewish families are waiting to be sent to concentration camps
**7**	00:58:01	01:59:27	Szpilman in a construction site
**8**	01:50:38	01:51:52	German soldiers are burning everything with flamethrower

## Appendix B

**Table A1:** Cross-validation selected features for valence (normalized count).

Single Channel Features	Channel Pairs	MSE
DiffEn_gammaAbs_F8 0.1 DiffEn_totalAbs_F8 0.08 Power_gammaRel_F3 0.08 Power_alphaRel_C4 0.06 DiffEn_thetaRel_F7 0.06 Power_thetaRel_C4 0.06 DiffEn_betaAbs_F3 0.04 DiffEn_alphaRel_C4 0.04 DiffEn_totalAbs_F7 0.04 Power_betaAbs_F3 0.02 Power_totalAbs_F4 0.02 Power_betaAbs_F7 0.02 DiffEn_deltaAbs_C4 0.02 Power_deltaRel_C4 0.02 Power_gammaAbs_F3 0.02 DiffEn_thetaRel_F3 0.02	DiffAsym_totalAbs_F8-F7 0.1 DiffAsym_deltaAbs_C4-C3 0.1 DiffAsym_totalAbs_F4-F3 0.1 DiffAsym_betaRel_C4-C3 0.1 RatAsym_gammaRel_C4-C3 0.1 RatAsym_betaRel_F8-F7 0.1 DiffAsym_thetaAbs_F4-F3 0.08 DiffAsym_betaAbs_F8-F7 0.08 DiffAsym_betaAbs_F4-F3 0.08 DiffAsym_deltaRel_F8-F7 0.06 DiffAsym_deltaRel_F4-F3 0.06 RatAsym_gammaRel_F4-F3 0.02 DiffAsym_betaAbs_C4-C3 0.02	multi_sample_entropy_S4 0.25 multi_renyi_entropy_S5 0.25 multi_renyi_entropy_S0 0.25 multi_sample_entropy_S0 0.25

**Table A2:** Cross-validation selected features for arousal (normalized count).

Single Channel Features	Channel Pairs	MSE
DiffEn_totalAbs_F7 0.1 DiffEn_totalAbs_F8 0.1 DiffEn_gammaAbs_F8 0.1 DiffEn_thetaAbs_F4 0.1 Power_gammaRel_F3 0.1 Power_alphaRel_C4 0.1 DiffEn_alphaRel_C4 0.1 DiffEn_deltaAbs_C4 0.08 Power_alphaRel_Fz 0.06 DiffEn_deltaAbs_F4 0.06 DiffEn_alphaRel_F7 0.04 Power_thetaRel_C4 0.02 DiffEn_thetaRel_C3 0.02 Power_deltaRel_C4 0.02	DiffAsym_thetaAbs_F4-F3 0.1 DiffAsym_deltaAbs_C4-C3 0.1 DiffAsym_totalAbs_F4-F3 0.1 DiffAsym_betaRel_C4-C3 0.1 DiffAsym_betaAbs_F4-F3 0.1 DiffAsym_totalAbs_F8-F7 0.1 DiffAsym_betaAbs_F8-F7 0.08 RatAsym_gammaRel_F4-F3 0.08 RatAsym_betaRel_F8-F7 0.08 DiffAsym_betaAbs_C4-C3 0.06 DiffAsym_deltaRel_F8-F7 0.04 RatAsym_gammaRel_C4-C3 0.04 DiffAsym_deltaRel_F4-F3 0.02	multi_sample_entropy_S4 0.25 multi_renyi_entropy_S5 0.25 multi_renyi_entropy_S0 0.25 multi_sample_entropy_S0 0.25
